# Evaluation of the Pharmacological Function of Ulinastatin in Experimental Animals

**DOI:** 10.3390/molecules17089070

**Published:** 2012-07-31

**Authors:** Chong-En Xu, Meng-Yuan Zhang, Cheng-Wei Zou, Ling Guo

**Affiliations:** 1 Department of Cardiac Surgery, Provincial Hospital Affiliated to Shandong University, 324# Jingwu-Weiqi Road, Jinan, 250021, China; 2 Department of Anesthesia, Provincial Hospital Affiliated to Shandong University, 324# Jingwu-Weiqi Road, Jinan, 250021, China; 3 Department of Cardiology, Provincial Hospital Affiliated to Shandong University, 324# Jingwu-Weiqi Road, Jinan, 250021, China

**Keywords:** oxidative injury, SOD, ulinastatin, AST, LDH, NO

## Abstract

Organ protection is a routine therapy in severe injuries. Our aim was to evaluate the beneficial effects of ulinastatin in experimental rats. Rats were randomly divided into a sham control, a model control and an ulinastatin-treated group. Malondialdehyde (MDA) and superoxide dismutase (SOD) levels were determined. Serum amylase, serum aspartate aminotransaminase (AST), lactate dehydrogenase (LDH) and creatine kinase isoenzyme (CKMD) activities, interleukin-8 (IL-8), tumor necrosis factor-α (TNF-α), nitric oxide (NO) and cardiac troponin I (nTnl) levels were examined. Results showed that ulinastatin decreased MDA levels and ameliorated the down-regulation of SOD activity. In addition, ulinastatin pretreatment may decrease serum AST, LDH and CKMD activities, IL-8, TNF-α, and nTnl levels, and enhance NO level. Our results demonstrated that oxidative injury occurred after IR and that ulinastatin exhibits significant protective effects against these effects.

## 1. Introduction

Despite significant improvements in medical and surgical management, coronary heart disease remains one of the major causes of morbidity and mortality in industrialized countries, with a large portion of patients suffering from myocardial infarction caused by ischemia and/or reperfusion injury [1]. Although treatment for ischemic heart disease such as revascularization therapy has progressed, it has been established that part of the cell death caused by transient coronary occlusion occurs at the time of reperfusion. The last two decades have witnessed major advances in understanding the mechanism of reperfusion injury, and this progress has allowed the use of different experimental strategies to decrease reperfusion injury [2].

Oxidative stress has been proposed as the unifying mechanism behind various risk factors of heart diseases [3] and it is implicated in many pathological disease states of the heart including hypertrophy [4], IR injury [5] and myocardial stunning [6]. Thus, a therapy that could address the high oxidative radical levels over a sustained period of time will have great potential in prevention of cardiac dysfunction. Ulinastatin, a multivalent enzyme inhibitor [7], is mainly used in treatment of pancreatitis and of acute circulatory failure due to severe infection. Ulinastatin has been used clinically for the prevention of multiple organ failure [8]. In recent years, ulinastatin has been found to display myocardial protective effects through increasing endogenous nitric oxide, inhibiting the expression of tumor necrosis factor and clearing away oxygen free radicals [9]. Wang and Zhang [10] reported that ulinastatin has dose-related protective effects on rat heart ischemia and reperfusion injury. Liu *et al*. [11] reported that ulinastatin can prevent acute inflammatory actions during liver transplantation. The present study aimed to investigate the preventive effects of ulinastatin in rats.

## 2. Results and Discussion

We examined the effects of ulinastatin on myocardial injury resulting from ischemia and reperfusion in rats assessed by biochemical evaluation. The main finding of this study indicates that ulinastatin provided marked cardioprotection in a well-characterized experimental model of myocardial IR injury in the rats.

Table 1 shows that left ventricular developed pressure (LVDP), left ventricular end-diastolic pressures (LVEDP), dp/dt_max_ and dp/dt_min_ were significantly decreased in IR group compared to sham group.

**Table 1:** Effect of ulinastatin on LVDP, LVEDP, dp/dt_max_ and dp/dt_min_ in three groups.

Group	LVDP (mm Hg)	LVEDP(mm Hg)	dp/dt_max_(mm Hg/s)	dp/dt_min_(mm Hg/s)
Sham	90.6 ± 7.3	1792.3 ± 164.3	1738.4 ± 180.2	1315.8 ± 155.2
IR	48.7 ± 3.5 **	992.1 ± 80.7 **	1203.7 ± 153.9 **	868.2 ± 74.2 **
IR + ulinastatin	87.2 ± 5.9 ^##^	1593.7 ± 138.1 ^##^	1682.8±174.2 ^##^	1301.4 ± 149.1 ^##^

** *P* < 0.01, compared with sham group; ^##^ *P* < 0.01, compared with IR group.

This indicated that heart contraction and stretching function in IR group rats were significantly decreased and obvious arrhythmia happened. Ulinastatin (10,000 u/kg) showed significantly enhanced LVDP, LVEDP, dp/dt_max_ and dp/dt_min_ in the IR + ulinastatin group compared to the sham group. These results suggested that ulinastatin (10,000 u/kg) pretreatment could improve heart function, strengthen heart contraction and stretching function in IR rats.

**Figure 1:**
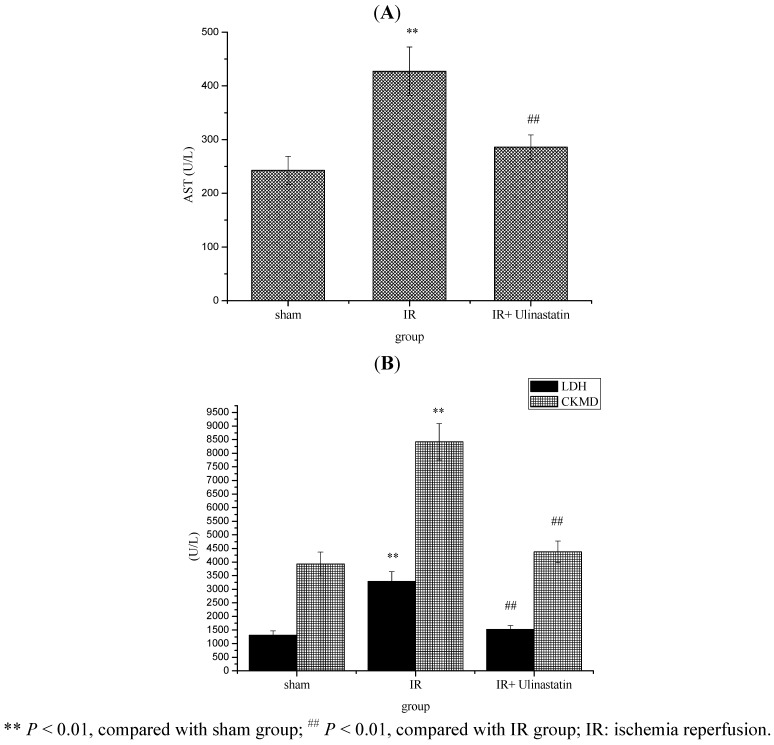
Effect of ulinastatin on serum AST (**A**) LDH and CKMD; (**B**) Activities in three groups.

Several biochemical markers, such as AST, CKMD and LDH were used to identify myocardial injury. Figure 1 shows that serum AST, LDH and CKMD activities were significantly increased in the IR group compared to the sham group. Ulinastatin (10,000 u/kg) significantly decreased the serum AST, LDH and CKMD activities in IR + ulinastatin group compared to sham group.

Serum aminotransferase activities have long been regarded as indicators of tissue injury. Injury of myocardium alters the structure and function of myocardium in rats, leading to leakage of enzymes from the cells. Therefore, the marked release of biochemical markers into the circulation indicates severe damage to heart tissue membranes during the reperfusion process [12]. Therefore, several biochemical markers, such as AST, CKMD and LDH were used to identify myocardial injury in our *in vivo* research experiment. It is confirmed that IR has a significant effect on the various membrane-bound enzymes in terms of increasing activities of plasma AST, CKMD and LDH [13]. The presence of ulinastatin protected myocardial cells against the harmful effects of IR by decreasing the leakage of the enzymes from the myocardial cells into the blood stream and maintaining the levels of these enzymes at normal values.

Some previous studies showed that myocardium IR may promote neutrophils to release a series of cytokines [14]. TNF-α could inhibit myocardium contractility, decrease ejection fraction and blood pressure. In addition, TNF-α could still stimulate endothelial and leucocyte cells to produce ET, NO and adhesion factor [15,16]. In microcirculation, TNF-α could make leucocyte adhere to endothelial cells, lead to severe microcirculatory disorders, promote intravascular coagulation, induce myocardial cell apoptosis, necrosis, and increase myocardial infarction area [17]. IL-8 could modulate polynuclear neutrophils adhesion, activation and transendothelial migration, and was an important inflammatory factor of myocardial IR injury. Its level was positively correlated with myocardial IR injury degree [18].

**Figure 2:**
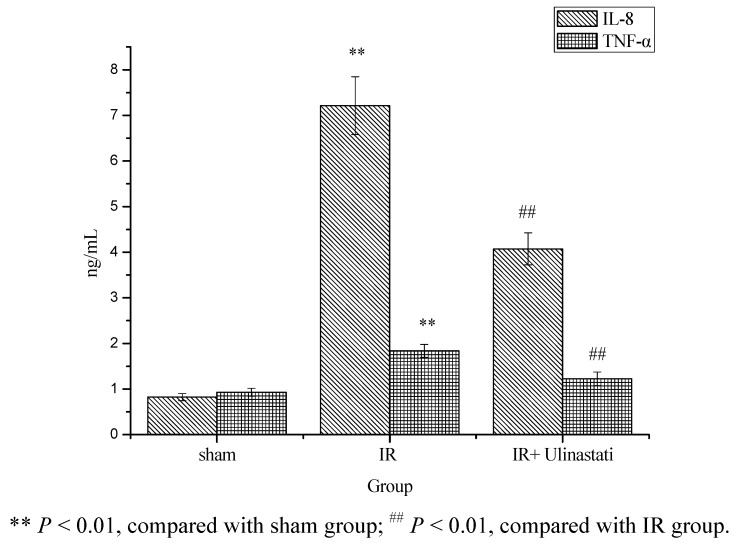
Effect of ulinastatin on serum IL-8 and TNF-α levels in three groups.

The result of serum IL-8 and TNF-α levels is presented in Figure 2. These results clearly indicated that IR resulted in a significant increase in the levels of serum IL-8 and TNF-α in IR group as compared to sham control. While, rats treated with ulinastatin (10,000 u/kg) showed significant decrease in serum IL-8 and TNF-α levels (*P* < 0.05) in IR + ulinastatin group as compared to IR control rats. The present results thus indicated that ulinastatin (10,000 u/kg) pretreatment may decrease the release of inflammatory mediators during IR and inhibit reperfusion myocardial cells apoptosis.

Nitric oxide (NO) plays multiple roles in the cardiovascular system mediating a number of physiological and pathophysiological processes. In smooth muscle cells, NO activates guanylate cyclase by hem-dependent mechanism resulting in increased concentration of guanosine 3′,5′-cyclic monophosphate (cGMP) that leads to a decreased intracellular concentration of Ca^2+^ and subsequent relaxation of the vessels [19]. Reduced basal availability of NO and impairment of endothelial NO-dependent mechanisms due to dysfunction of the normally protective endothelium may be involved in the pathogenesis of several cardiovascular diseases including atherosclerosis, hypertension, heart failure, coronary heart disease, arterial thrombotic disorders, and stroke [20]. The cardiac troponins I (cTnI) has excellent sensitivity and specificity and are superior to CK-MB in indicating minor myocardial injury. Troponin I, prevents the contraction of muscle in the absence of calcium and Troponin C [21,22].

**Figure 3:**
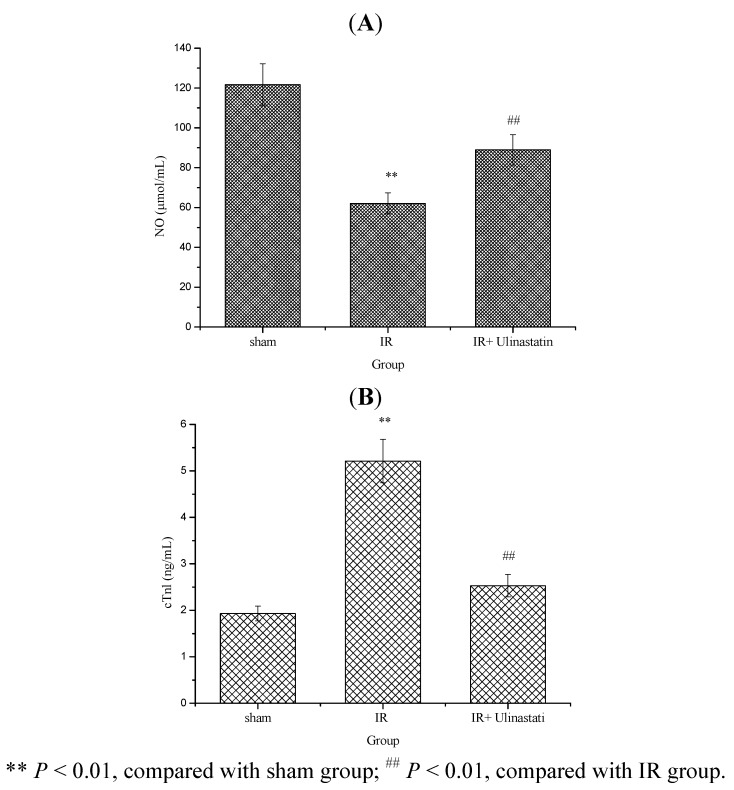
Effect of ulinastatin on serum NO and nTnl levels in three groups.

The serum NO and cTnl level results are presented in Figure 3. These results clearly indicated that IR resulted in a significant decrease in serum NO level and increase in cTnl level in IR group as compared to sham control, while rats treated with ulinastatin (10,000 u/kg) showed a significant increase in serum NO and decrease in serum cTnl levels (*P* < 0.05) in IR + ulinastatin group as compared to IR control rats. 

Increasing evidences show that oxidative stress causes the overgeneration and accumulation of ROS (superoxide anion and hydroxyl radicals and other reactive species) that are central to myocardial ischemia injury in myocardial ischemia [23]. The generation of ROS is a part of normal cell metabolism and defense, cellular systems also produce antioxidant defenses against ROS. Myocardial ischemia causes alterations in the defense mechanism against oxygen free radicals (OFRs), which play central roles in cardiac physiology and pathophysiology. It is now well established that when the production of ROS exceeds the capacity of antioxidant defenses, and at last brings about oxidative damage of membrane lipids, proteins, carbohydrates and DNA [24,25,26]. Accordingly, stopping overgeneration and accumulation of ROS is an important means for the prevention and treatment of ischemic heart diseases. In addition, ulinastatin has been shown to inhibit oxidative stress and acts as an effective reactive oxygen species scavenger during myocardial ischemia and reperfusion [27]. We also found that ulinastatin could decrease the level of MDA and increase the level of SOD.

**Figure 4:**
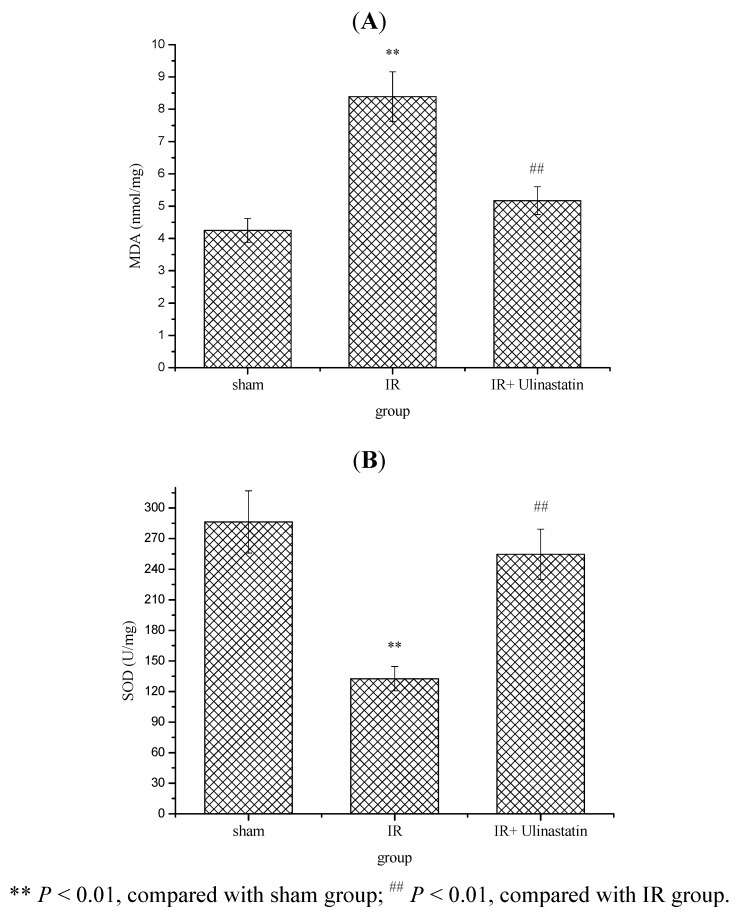
Effect of ulinastatin on myocardium MDA level and SOD activity in three groups.

The present results revealed that IR produced a statistically significant increase (*P* < 0.01) in myocardium MDA level and decrease in SOD activity in IR rats compared to sham rats (Figure 4).

**Figure 5:**
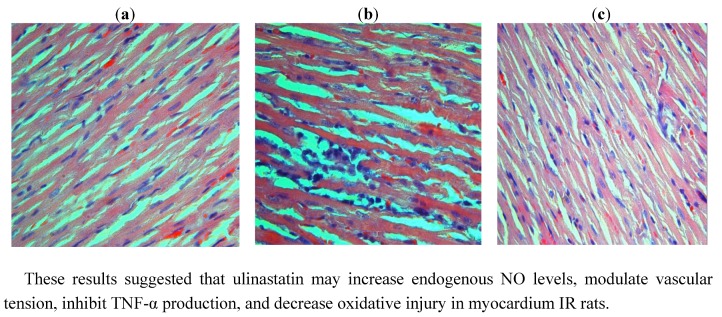
Histopathological changes in the myocardium at three groups. (**a**) The sham group, with a well-arranged cardiac cells and integrated membrane. (**b**) The IR group, showing swelling myocardial cells, a disordered striated cardiac muscle, and local myocardial necrosis. A great number of erythrocytes are present and local infiltration of inflammatory cells is observed. (**c**) The IR + ulinastatin group, showing a well-arranged cardiac cells and a tiny amount of neutrophil infiltration.

Administration of ulinastatin (10,000 u/kg) significantly decreased myocardium MDA level and increased SOD activity in IR + ulinastatin group compared to IR group. In the sham group, no infiltration of inflammatory cells was found, myocardial fibers were regularly arranged, and the cell border was intact. In IR group, IR caused myocardial fibers twist, broken and even necrotic, myocardial cells swollen and degenerated, cell nucleus concentrated or dissolved, and neutrophil infiltrated. In IR + ulinastatin group, few neutrophil infiltration and relative integrated structure were seen (Figure 5).

These results suggested that ulinastatin may increase endogenous NO levels, modulate vascular tension, inhibit TNF-α production, and decrease oxidative injury in myocardium IR rats.

## 3. Experimental

### 3.1. Animals

A randomized and blinded study was conducted using adult Sprague-Dawley rats (obtained from Animal center of Shandong University, Jinan, China) weighing 260 ± 10 g. Rats were divided into three groups (sham, IR and IR + ulinastatin groups). Each group contained fifteen rats. While one group was subjected to sham surgery, the other two groups received IR surgery (30 min coronary artery ligation followed by reperfusion), with or without the intravenous injection of 100 mL of saline containing ulinastatin (10,000 u/kg), 5 min before ischemia. The animal studies were approved by Shandong University Institutional Animal Care and Use Committee and by the Institutional Ethics Committee on Animal Research of our Hospital.

### 3.2. The IR Model and Heart Tissue Collection

Rats were anesthetized with sodium pentobarbital (30 mg kg^−1^ BW^−1^). Rats were shaven, and an animal respiratory ventilator maintained the artificial respiratory rate at 60/min and the tidal volume at 7–10 mL. A left thoracotomy was performed between the 3rd and 4th rib. The heart was almost fully exposed and the pericardium was cleaned. A 4/0 silk suture was attached to a 10 mm micropoint reverse-cutting needle and quickly placed under the left anterior descending coronary artery in the Sham group. In the other groups, a small plastic snare was threaded through with 4/0 silk suture, and placed in contact with the heart. The artery could then be occluded by applying tension to the silk suture for 30 min, and reperfused for 120 min by releasing the tension prior to the completion of the experiment. The chest cavity was then closed, and the rats were monitored in the animal facility for 120 min. After 120 min of reperfusion, blood was taken from the abdominal aorta to determine serum CK and AST levels, which were indicative of heart function. Rats were then sacrificed by cervical dislocation, and the hearts were rapidly removed, placed in ice-cold physiological saline, cleaned of blood, snap-frozen in liquid nitrogen, and stored in −80 °C.

### 3.3. Hemodynamic Measurements

After end of the 2 h reperfusion period, rats were re-anesthetized and cardiac function was analysed using 2 F Millar catheter (Millar Instruments, Houston, TX, USA). The left ventricular diastolic pressure (LVDP), LV end-diastolic pressure (LVEDP), was digitally processed using a hemodynamic analysing system (Power lab Hardware; AD Instruments, Charlotte, NC, USA). Maximal positive and negative values of the instantaneous first derivative of LV pressure (+dp/dt_max_ and −dp/dt_max_) were measured by computer algorithms.

### 3.4. Biochemical Parameters

Serum AST, LDH and CKMD activities and NO, cTnl levels were measured with appropriate enzyme kits (NanJing JianChen Bioengineering Ltd, NanJing, China). Serum IL-8 and TNF-α levels were measured with radioimmunoassay kits (NanJing JianChen Bioengineering Ltd, NanJing, China).

Lipid peroxidation was estimated by measuring thiobarbituric acid-reactive substances (TBARS) and expressed in terms of malondialdehyde (MDA) content, according to the method of Draper and Hadley [28]. The MDA values were calculated using 1,1,3,3-tetraethoxypropane as standard and expressed as nmol of MDA/mg.

The activity of superoxide dismutase (SOD) was assayed by monitoring its ability to inhibit the photochemical reduction of nitro blue tetrazolium (NBT). Each 1.5 mL reaction mixture contained 100 mM Tris/HCl (pH 7.8), 75 mM NBT, 2 μM riboflavin, 6 mM EDTA, and 200 μL of supernatant. Monitoring the increase in absorbance at 560 nm followed the production of blue formazan. One unit of SOD is defined as the quantity required to inhibit the rate of NBT reduction by 50% as described by Winterbourn *et al.* [29].

### 3.5. Histological Studies

Hearts from the normal and experimental rats were fixed in 10% buffered formalin and were processed for paraffin sectioning. Sections of about 5 μm thickness were stained with hematoxylin and eosin to evaluate under light microscope.

### 3.6. Statistical Analysis

Results are expressed as mean ± S.E.M. One-way analysis of variance (ANOVA) followed by Dunnett’s test was used for statistical evaluation. *P* values less than 0.05 were considered significant.

## 4. Conclusion

In the present study, ulinastatin can improve heart function, strengthen heart contraction and stretching function by enhancing the LVDP, LVEDP, dp/dt_max_ and dp/dt_min_ in IR rats. In addition, ulinastatin displayed better effects on inhibiting the activities of AST, CK and LDH. Furthermore, ulinastatin can enhance immunity and antioxidant activities in myocardium IR rats. At last, histological examination further confirms that ulinastatin at selected dose may decrease myocardium IR-induced injury in experimental rats. These observations indicated that ulinastatin may represent a potential protective agent for some heart diseases. 
